# Interpretable machine-learning for depression classification based on a three-component EEG marker

**DOI:** 10.3389/fncom.2026.1869159

**Published:** 2026-08-05

**Authors:** Haisheng Zhang, Jing Kan, Wei Tong, Bicheng Wu, Ye Zhao, Yongchun Ma

**Affiliations:** 1 Department of Clinical Psychology, Affiliated Hangzhou First People's Hospital, School of Medicine, Westlake University, Hangzhou, Zhejiang, China; 2 Advanced Institute of Information Technology (AIIT), Peking University, Hangzhou, Zhejiang, China; 3 School of Computer Science and Technology, Hangzhou Dianzi University, Hangzhou, Zhejiang, China; 4 Tongde Hospital of Zhejiang Province, Hangzhou, Zhejiang, China

**Keywords:** electroencephalography, frontal asymmetry, functional connectivity, interpretable machine-learning, major depressive disorder, SHAP

## Abstract

Electroencephalography (EEG)-based depression classification requires interpretable machine-learning approaches and validation strategies that avoid subject-level information leakage. This single-center pilot study developed and internally evaluated a marker-based interpretable machine-learning framework using eight-channel resting-state EEG. After quality control, 48 participants were included, comprising 23 clinician-diagnosed major depressive disorder (MDD) patients recruited at Zhejiang Provincial Tongde Hospital and 25 healthy controls (HCs). Subject-level spectral, entropy/complexity, asymmetry, and coherence-based connectivity features were extracted from pre-processed EEG epochs. Exploratory feature analysis was used to define a three-component EEG marker comprising fronto-posterior beta- and gamma-band coherence heterogeneity and F8–F7 beta-band asymmetry variability. The resulting marker was evaluated using classical classifiers under strict subject-wise leave-one-subject-out validation and compared with EEGNet and 1D-CNN baselines under the same subject-wise protocol. Among the evaluated classical models, RBF-SVM achieved the highest subject-level discrimination in the primary native-reference 2-s analysis, with an AUC of 0.910, accuracy of 85.42%, sensitivity of 82.61%, and specificity of 88.00%. The primary result used the native A1/A2 acquisition reference and non-overlapping 2-s epochs, consistent with segmentation used in previous resting-state EEG depression studies. SHAP analysis indicated that fronto-posterior gamma-band coherence heterogeneity and F8–F7 beta-band asymmetry variability were the dominant contributors to the RBF-SVM decision function. These findings support the exploratory value of compact EEG markers for interpretable internal discrimination in data-limited eight-channel settings and motivate validation in independent external cohorts.

## 1 Introduction

Depressive disorders, including major depressive disorder (MDD), are among the most prevalent and disabling mental disorders worldwide. Recent estimates based on the Global Burden of Disease 2021 database indicate that depressive disorders affected more than 330 million people globally in 2021 (Zhou et al., 2025). Clinical assessment of MDD continues to rely primarily on symptom-based interviews and self-report scales, which are central to diagnosis but remain inherently subjective and provide limited access to the underlying neurophysiology. There is therefore continued interest in quantitative, brain-based measures that could complement clinical evaluation. Electroencephalography (EEG) is particularly attractive for this purpose because it is non-invasive, relatively low-cost, portable, and widely available. Resting-state EEG captures spontaneous cortical activity related to affective and cognitive regulation, making it a suitable modality for neurophysiological studies of depression (Newson and Thiagarajan, 2019; Heitmann et al., 2026).

Resting-state EEG studies have reported a range of depression-related alterations, which can be broadly grouped into spectral, asymmetry, complexity, and connectivity measures. Changes in band-specific power have been described, with recent evidence suggesting increased beta activity as one of the more consistent resting-state EEG findings in depression (Heitmann et al., 2026). Frontal alpha asymmetry is among the most extensively studied candidate EEG markers of depression vulnerability and affective regulation, with heterogeneous findings across studies (Allen and Reznik, 2015; Luo et al., 2025). Measures of signal complexity and irregularity have likewise been used to characterize altered cortical dynamics in depression. Beyond region- or channel-level descriptors, MDD is increasingly viewed in terms of distributed network dysfunction, and EEG functional connectivity studies have reported altered coordination between frontal and posterior regions (Leuchter et al., 2012; Miljevic et al., 2023). Together, these lines of evidence motivate features that capture frontal asymmetry, fronto-posterior connectivity, and signal complexity, with the present marker focusing specifically on fronto-posterior coherence heterogeneity and frontotemporal asymmetry variability.

Recent EEG-based depression classification studies have increasingly explored deep and spatiotemporal architectures, including CNN–GRU models based on spatiotemporal EEG representations, weighted GCN–GRU models, graph convolutional networks incorporating attention mechanisms and subject partitioning, and transformer-based temporal–frequency–spatial modeling (Liu et al., 2022; Wang et al., 2024; Zhang et al., 2024b; Xu et al., 2026). These studies demonstrate the potential of advanced representation learning for EEG-based depression recognition. However, their reported performance also underscores the importance of rigorous validation, because EEG datasets are often small and segmented into multiple epochs per participant. Despite encouraging classification results, several methodological concerns therefore remain central to the interpretation of EEG-based studies of depression. First, performance is often estimated by randomly splitting EEG epochs into training and test sets. Because multiple epochs from the same participant can then appear in both sets, models may exploit subject-specific signal characteristics rather than disorder-related patterns, leading to substantial overestimation of performance on unseen subjects (Brookshire et al., 2024). Subject-wise validation, in which all recordings from a held-out participant are excluded from training, is therefore necessary for valid subject-level performance estimation, yet it is still not consistently adopted. Second, end-to-end deep-learning models, although increasingly applied to EEG, can be difficult to train robustly when the number of participants is small: even when many epochs are available, the number of statistically independent units remains limited by the number of subjects, leaving such models prone to overfitting and poor cross-subject generalization (Roy et al., 2019; Xu et al., 2020). Third, high-dimensional feature pipelines and end-to-end models may offer limited interpretability, often reporting predictive accuracy without clarifying which neurophysiological patterns drive the decisions. These issues motivate a validation framework that treats the participant as the unit of analysis and emphasizes compact, interpretable EEG markers in data-limited settings.

In this study, we used a small single-center eight-channel resting-state EEG cohort to develop and internally evaluate an interpretable machine-learning framework for depression-related group discrimination based on a three-component EEG marker. We first extracted a broad set of subject-level features spanning spectral power, entropy/complexity, frontal asymmetry, and coherence-based fronto-posterior connectivity. Exploratory feature analysis was then used to identify group-related patterns supported by statistical evidence, discriminative ability, resampling stability, and neurophysiological plausibility. Based on this convergent exploratory evidence, we defined a compact three-component EEG marker consisting of fronto-posterior beta/gamma coherence heterogeneity and F8–F7 beta-band asymmetry variability. The marker was evaluated with several classical classifiers under strict subject-wise leave-one-subject-out (LOSO) validation, ensuring that all data from each held-out participant were excluded from feature standardization and model fitting. To provide a reference against end-to-end alternatives, we also evaluated EEGNet and 1D-CNN baselines under the same subject-wise protocol. Finally, we applied SHAP analysis to interpret the marker-based classifier. Overall, this pilot study was designed to evaluate whether a compact EEG marker could support interpretable internal discrimination in a data-limited setting while reducing the risk of leakage-driven performance inflation.

## 2 Materials and methods

**Figure 1:**
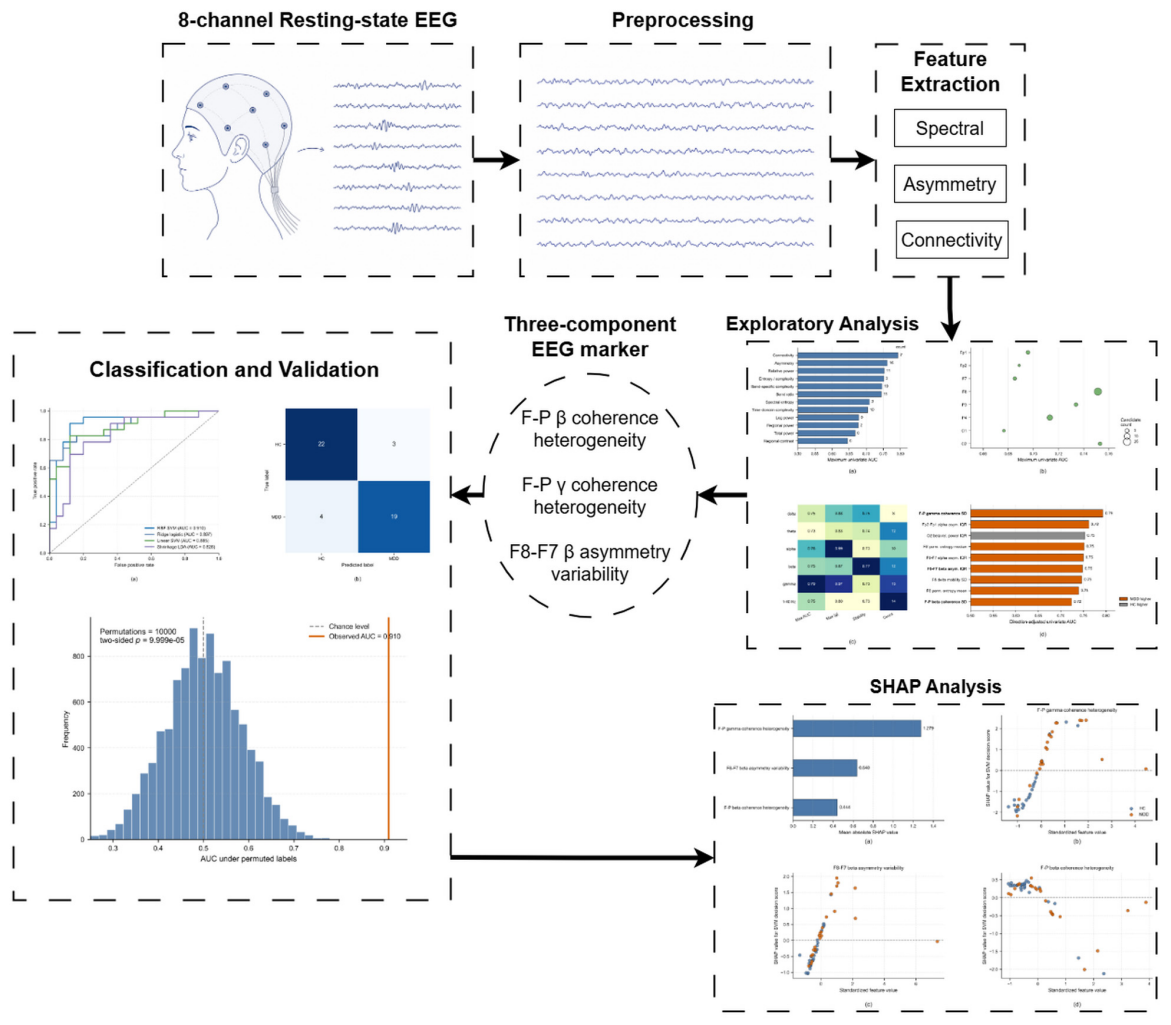
Overview of the study framework. Eight-channel resting-state EEG signals were pre-processed and transformed into subject-level spectral, asymmetry, and connectivity features. Exploratory feature analysis was then used to define a compact three-component EEG marker, which was subsequently evaluated under subject-wise LOSO validation and interpreted using SHAP analysis.

This section describes the overall analytical framework used to develop, validate, and interpret the proposed EEG marker for MDD classification (Figure 1). The analysis was based on a clinical eight-channel resting-state EEG cohort. Briefly, the recordings were pre-processed and transformed into subject-level features, from which a compact three-component marker was defined through exploratory feature analysis. The marker was then evaluated under strict subject-wise leave-one-subject-out validation and interpreted using SHAP analysis. Throughout the study, the participant was treated as the unit of analysis to reduce information leakage from within-subject epoch dependence.

### 2.1 Dataset and participants

This study used a self-collected Zhejiang Provincial Tongde Hospital dataset (ZJTD dataset), an eight-channel resting-state EEG dataset acquired from patients with clinically diagnosed major depressive disorder (MDD) and healthy controls (HCs). Participants were recruited at Zhejiang Provincial Tongde Hospital, China, and the MDD diagnosis was established by a chief physician in the hospital's clinical outpatient service before EEG acquisition. The original cohort included 24 individuals in the MDD group and 26 HCs. The PHQ-9 and GAD-7 scales were used to quantitatively characterize depressive and anxiety symptom burden. The study was conducted in accordance with the Declaration of Helsinki and approved by the Ethics Committee of Zhejiang Provincial Tongde Hospital (Approval No.: ZTDLL-2025-128-JY; Approval Date: March 13, 2025). All participants were informed of the study purpose, procedures, and potential risks, and written informed consent was obtained before data collection. Demographic characteristics of the original ZJTD cohort are summarized in Table 1; the MDD and HC groups did not differ significantly in age or sex distribution.

**Table 1:** Demographic characteristics of the original ZJTD cohort.

Characteristic	MDD (*n* = 24)	HC (*n* = 26)	*p*-value
Age (years)	37.25 ± 21.35	31.34 ± 15.14	0.269
Sex (male/female)	10/14	9/17	0.772

Values are presented as mean ± standard deviation for age and counts for sex. The *p*-value for age was calculated using Welch's two-sample *t*-test, and the *p*-value for sex distribution was calculated using Fisher's exact test. Demographic characteristics are reported for the original recruited cohort; two participants were subsequently excluded during EEG quality control, yielding the final analysis cohort of 48 participants (23 MDD and 25 HCs).

EEG data were acquired during a resting-state recording using a non-invasive dry-electrode system developed by Shunao (Hangzhou) Intelligent Technology Co., Ltd., at a sampling rate of 250 Hz. During acquisition, bilateral A1/A2 mastoid reference electrodes were used as the recording reference. Raw BDF files contained eight EEG channels labeled EEG_1–EEG_8, which were mapped in fixed order to the international 10–20 positions Fp1, Fp2, F7, F8, P3, P4, O1, and O2. The low-channel montage sampled bilateral homologous frontopolar, frontotemporal, parietal, and occipital electrodes and supported the predefined analyses of left–right asymmetry, posterior rhythmic activity, and fronto-posterior sensor-pair interactions.

### 2.2 EEG pre-processing

All recordings were processed with an identical pipeline prior to feature extraction and classification. Raw BDF files were loaded, and the eight EEG channels (Fp1, Fp2, F7, F8, P3, P4, O1, and O2) were extracted in a fixed channel order and represented in microvolts. For each participant, the continuous signals were demeaned channel-wise and band-pass filtered with a fourth-order zero-phase Butterworth filter (1–40 Hz) to retain conventional EEG rhythms while attenuating slow drifts and high-frequency noise; no polynomial detrending or baseline correction was applied. No re-referencing was performed, and the native bilateral A1/A2 mastoid recording reference was retained for the primary analysis. The filtered signals were segmented into non-overlapping 2-s epochs, a length chosen to provide sufficient samples for spectral and connectivity estimation while preserving an adequate number of epochs per participant for subject-level aggregation. Epoch-level quality control was then applied: an epoch was rejected if it contained non-finite values or flat channels, if its maximum absolute amplitude exceeded 500 μV, if its peak-to-peak amplitude exceeded 1,000 μV, or if its robust outlier score for epoch-level signal energy and peak-to-peak variation exceeded 6.0. After artifact rejection, participants retaining fewer than 30 artifact-free epochs were excluded from all subsequent analyses. The retained epochs formed the basis for all downstream analyses. For feature-based modeling, epoch-level measurements were aggregated within each participant into subject-level features. For the deep-learning baselines, models operated on individual epochs, and epoch-level predicted probabilities were averaged into a single subject-level prediction. In all analyses, the participant was the unit of analysis.

### 2.3 Reference and epoch-length considerations

The primary analysis retained the native bilateral A1/A2 mastoid acquisition reference and non-overlapping 2-s epochs. Previous resting-state EEG depression studies have used 2-s segments for artifact rejection and spectral or connectivity estimation (Huang et al., 2023; Zhang et al., 2024a). For coherence estimation, the full 2-s epoch corresponded to a frequency spacing of 0.5 Hz. Within the analyzed ranges, each epoch contained approximately 26–60 beta cycles (13–30 Hz) and 60–80 gamma cycles (30–40 Hz), supporting band-level beta/gamma estimation while retaining sufficient artifact-free segments for participant-level aggregation. Welch power-spectral estimation used a Hann window, 1-s segments, and 50% overlap, corresponding to a 1-Hz frequency grid.

### 2.4 Feature extraction

After pre-processing, features were extracted from the retained artifact-free 2-s epochs. For each participant, candidate features were computed at the epoch level or within predefined channel groups and then summarized into subject-level descriptors. The initial feature space was designed to characterize spectral activity, signal irregularity, hemispheric asymmetry, and fronto-posterior functional connectivity, and served as the input to the subsequent exploratory analysis and marker definition described in Section 2.5. The mathematical expressions for all extracted features are given in Equations (1)–(4).

#### 2.4.1 Spectral power and band-ratio features

For each epoch and channel, spectral power was estimated in five conventional frequency bands: delta (1–4 Hz), theta (4–8 Hz), alpha (8–13 Hz), beta (13–30 Hz), and gamma (30–40 Hz), using Welch spectral estimation with a Hann window, *n*_perseg_ equal to the smaller of 1 s of data and the epoch length, 50% overlap, constant detrending, and density scaling (Welch, 1967; Newson and Thiagarajan, 2019). Let *P*_*e, c, b*_ denote the band power of epoch *e*, channel *c*, and band *b*. Both absolute and relative band power were extracted, with relative power defined as


$$\begin{array}{l} R_{e,c,b}=\frac{P_{e,c,b}}{\sum_{b^{\prime }\in B}P_{e,c,b^{\prime }}}, \end{array}$$ (1)

where B is the set of frequency bands. To characterize the balance between slow and fast oscillatory components, several band-ratio features were also computed, including theta/beta, alpha/beta, theta/alpha, and slow/fast power ratios. These descriptors were included because alterations in rhythmic balance and frequency-specific activity have been widely associated with affective and cognitive brain states (Newson and Thiagarajan, 2019; Heitmann et al., 2026).

#### 2.4.2 Entropy and complexity features

Entropy and complexity features were extracted to describe EEG irregularity and temporal complexity, comprising fuzzy entropy, permutation entropy, differential entropy, spectral entropy, and Hjorth parameters (Chen et al., 2007; Bandt and Pompe, 2002; Duan et al., 2013; Inouye et al., 1991; Hjorth, 1970). Fuzzy entropy quantified the similarity and regularity of short EEG segments, whereas permutation entropy characterized the complexity of ordinal temporal patterns. Differential entropy was used as a logarithmic energy-related descriptor under a Gaussian approximation (Cover and Thomas, 2006; Duan et al., 2013); for a segment with variance σ^2^,


$$\begin{array}{l} \text{DE}=\frac{1}{2}\log\left(2\pi \;e\sigma^{2}\right). \end{array}$$ (2)

Spectral entropy was computed from the normalized power distribution across frequency components to describe the disorder of the spectral profile, and Hjorth mobility and complexity summarized signal variation and waveform complexity in the time domain. These features were computed for the available channels and frequency-specific components where applicable.

#### 2.4.3 Asymmetry and connectivity features

Asymmetry features were computed from log-transformed band-power differences between frontal channel pairs to capture lateralized frontal activity (Allen and Reznik, 2015; Luo et al., 2025). Two right–left pairs were considered, Fp2–Fp1 and F8–F7; for a pair (*c*_*R*_, *c*_*L*_), the band-specific asymmetry was defined as


$$\begin{array}{l} A_{e,\left(c_{R},c_{L}\right),b}=\log\left(P_{e,c_{R},b}\right)-\log\left(P_{e,c_{L},b}\right), \end{array}$$ (3)

where *b* denotes the frequency band. These descriptors characterized frontal and frontotemporal lateralization.

Functional connectivity was quantified using magnitude-squared coherence, a standard frequency-domain measure of linear association between two signals (Welch, 1967; Leuchter et al., 2012; Miljevic et al., 2023). For channels *i* and *j*, coherence at frequency *f* was defined as


$$\begin{array}{l} C_{ij}\left(f\right)=\frac{|S_{ij}\left(f\right){|}^{2}}{S_{ii}\left(f\right)S_{jj}\left(f\right)}, \end{array}$$ (4)

where *S*_*ij*_(*f*) is the cross-spectral density between the two channels and *S*_*ii*_(*f*), *S*_*jj*_(*f*) are the corresponding auto-spectral densities. Band-specific coherence was obtained by summarizing coherence values within each frequency band. For the fronto-posterior coherence features used in the marker, cross-spectral quantities were estimated from Hanning-windowed epoch-level Fourier coefficients over the full 2-s epoch, giving 0.5-Hz frequency spacing. Connectivity analysis focused on predefined region-level interactions between the frontal set F={Fp1,Fp2,F7,F8} and the posterior set P={P3,P4,O1,O2}.

#### 2.4.4 Subject-level aggregation

Most features were first computed at the epoch level and then summarized within each participant to obtain subject-level descriptors. For each epoch-level feature, the within-subject distribution across retained epochs was summarized using the mean, median, standard deviation, and interquartile range, capturing both the central tendency and the within-subject variability of EEG-derived measurements.

For connectivity features, pairwise coherence values were first computed for predefined channel-pair sets within each frequency band. In particular, fronto-posterior connectivity was characterized using coherence values across frontal–posterior channel pairs, which were then summarized by their mean and standard deviation within each band.

The subject-level feature matrix therefore contained both temporal summaries across retained epochs and spatial summaries across channel pairs. This aggregation strategy allowed the subsequent analyses to evaluate not only the average spectral, asymmetry, entropy, and connectivity levels, but also their within-subject variability and spatial heterogeneity.

### 2.5 Exploratory feature analysis

#### 2.5.1 Subject-level feature comparison

To examine group-level differences between patients with MDD and HCs, exploratory comparisons were performed on the subject-level features described in Section 2.4, which served as the units of statistical analysis. The relevant statistical calculation formulas are provided in Equations (5)–(12). For each candidate feature, let ${\left\{x_{i}^{\left(1\right)}\right\}}_{i=1}^{n_{1}}$ and ${\left\{x_{j}^{\left(0\right)}\right\}}_{j=1}^{n_{0}}$ denote the feature values in the MDD and HC groups, with *n*_1_ and *n*_0_ subjects respectively. A two-sided Mann–Whitney *U* test was used (Mann and Whitney, 1947). After ranking all *n*_1_+*n*_0_ observations jointly, the *U* statistic for the MDD group was


$$\begin{array}{l} U_{1}=R_{1}-\frac{n_{1}\left(n_{1}+1\right)}{2}, \end{array}$$ (5)

where *R*_1_ is the rank sum of the MDD group, and the corresponding statistic for the HC group was


$$\begin{array}{l} U_{0}=n_{1}n_{0}-U_{1}. \end{array}$$ (6)

The test statistic *U* = min(*U*_1_, *U*_0_) yielded a two-sided *p* value for each feature. This non-parametric test was chosen for the limited sample size and its robustness to non-Gaussian EEG-derived feature distributions. The resulting nominal *p* values served as exploratory indicators of group-level differences and were considered jointly with effect size, univariate discrimination, resampling stability, and neurophysiological interpretability, as described below.

#### 2.5.2 Effect size and univariate discrimination

Effect size and univariate discriminative ability were computed to complement the group-comparison statistics with measures of magnitude and classification relevance. The standardized group difference was quantified using Hedges' *g*, a small-sample correction of Cohen's *d* (Hedges, 1981). Let ${\bar{x}}_{1}$, ${\bar{x}}_{0}$ denote the group means and $s_{1}^{2}$, $s_{0}^{2}$ the group variances; the pooled standard deviation was


$$\begin{array}{l} s_{p}=\sqrt{\frac{\left(n_{1}-1\right)s_{1}^{2}+\left(n_{0}-1\right)s_{0}^{2}}{n_{1}+n_{0}-2}}, \end{array}$$ (7)

the uncorrected standardized difference was


$$\begin{array}{l} d=\frac{{\bar{x}}_{1}-{\bar{x}}_{0}}{s_{p}}, \end{array}$$ (8)

and Hedges' *g* was


$$\begin{array}{l} g=Jd,\;J=1-\frac{3}{4\left(n_{1}+n_{0}\right)-9}, \end{array}$$ (9)

where *J* is the finite-sample correction factor. A positive *g* indicates larger values in the MDD group and a negative *g* larger values in the HC group.

Single-feature discriminative ability was assessed by univariate ROC analysis, treating MDD subjects as the positive class. The univariate AUC can be interpreted as the probability that a randomly selected positive subject has a higher score than a randomly selected negative subject, with ties counted as one half (Hanley and McNeil, 1982). Specifically,


$$\begin{array}{l} \text{AUC}=\mathrm{Pr}\left(X_{1}>X_{0}\right)+\frac{1}{2}\mathrm{Pr}\left(X_{1}=X_{0}\right), \end{array}$$ (10)

where *X*_1_ and *X*_0_ are feature values from randomly selected MDD and HC subjects. Because some features take lower values in the MDD group, a direction-adjusted univariate AUC was defined as


$$\begin{array}{l} \text{AU}{\text{C}}_{\text{best}}=\max\left\{\text{AUC},1-\text{AUC}\right\}, \end{array}$$ (11)

and the corresponding direction was recorded. These direction-adjusted univariate AUC values summarized the exploratory discriminative potential of individual features. Final classifier performance was assessed under subject-wise validation (Section 2.6).

#### 2.5.3 Feature prioritization

Candidate features were then prioritized for exploratory interpretation and low-dimensional marker definition by combining statistical, discriminative, stability, and neurophysiological evidence. The robustness of each feature's group-difference direction was assessed by bootstrap resampling (Efron, 1979). In each iteration, subjects were resampled within the MDD and HC groups and the direction was recomputed; direction stability was defined as the proportion of iterations preserving the dominant direction:


$$\begin{array}{l} \text{Stability}=\max\left\{\frac{1}{B}\overset{B}{\underset{b=1}{\sum }}I\left(d_{b}=1\right),\frac{1}{B}\overset{B}{\underset{b=1}{\sum }}I\left(d_{b}=-1\right)\right\}, \end{array}$$ (12)

where *B* is the number of bootstrap iterations, *d*_*b*_ the direction in the *b*-th sample, and *I*(·) the indicator function; a value near 1 indicates a consistent direction under resampling.

Prioritization jointly considered the nominal group-comparison *p* value, absolute effect size |*g*|, direction-adjusted univariate AUC AUC_best_, bootstrap direction stability, and neurophysiological interpretability. Those showing a consistent direction, moderate effect size, non-trivial univariate discrimination, and a plausible link to depression-related EEG mechanisms were regarded as more informative for subsequent modeling. This process organized candidate features across families, channels, and frequency bands and informed the three-component marker definition.

### 2.6 Classification and validation

#### 2.6.1 Subject-wise validation

All models were evaluated under subject-wise LOSO validation. In each outer fold, one participant was held out as the test subject while all remaining participants were used for model fitting and validation; this was repeated until every participant had served once as the held-out subject. The LOSO design prevents the information leakage that arises when epochs from the same participant appear in both training and test sets, a known source of inflated performance estimates in EEG-based classification studies (Brookshire et al., 2024). In every fold, all epochs and subject-level features from the held-out participant were excluded from model fitting, feature standardization, validation, and early stopping, so that final predictions were always obtained for a participant entirely unseen during training. Continuous features were standardized within each fold using the mean and standard deviation of the training subjects only, which were then applied to the held-out subject.

For probability-based classifiers, predicted probabilities were converted to class labels at a fixed threshold of 0.5. For SVM models, which produce decision scores rather than probabilities, the default threshold of 0 was used, with positive scores classified as MDD and negative scores as HC. The continuous subject-level scores were retained for AUC computation. Calculations of classification evaluation metrics are defined in Equations (13)–(21).

#### 2.6.2 Marker-based classical classifiers

The compact subject-level marker determined from the exploratory analysis was used as the common input to a set of classical classifiers for MDD vs. HC classification. The marker was represented as a low-dimensional feature vector $x_{i}\in {\mathbb{R}}^{d}$ for participant *i*, where *d* is the number of marker components, and all classifiers were trained and evaluated at the subject level.

Four classifier families were compared on this common input: ridge logistic regression, linear support vector machine (linear SVM), shrinkage linear discriminant analysis, and radial-basis-function support vector machine (RBF-SVM) (Hoerl and Kennard, 1970; Cortes and Vapnik, 1995; Ledoit and Wolf, 2004; Pedregosa et al., 2011). Ridge logistic regression served as an interpretable linear classifier suited to small-sample settings; for participant *i*, the predicted probability of MDD was


$$\begin{array}{l} {\hat{p}}_{i}=\mathrm{Pr}\left(y_{i}=1|x_{i}\right)=\frac{1}{1+\exp\left[-\left(\beta_{0}+x_{i}^{⊤}\beta \right)\right]}, \end{array}$$ (13)

with *y*_*i*_ = 1 for MDD and *y*_*i*_ = 0 for HC. The parameters were estimated by minimizing the *L*_2_-penalized negative log-likelihood


$$\begin{array}{l} L\left(\beta_{0},\beta \right)=-\underset{i}{\sum }\left[y_{i}\log\left({\hat{p}}_{i}\right)+\left(1-y_{i}\right)\log\left(1-{\hat{p}}_{i}\right)\right]+\lambda \|\beta {\|}_{2}^{2}, \end{array}$$ (14)

where the penalty stabilizes estimation. Linear SVM and shrinkage LDA evaluated linear decision boundaries, whereas RBF-SVM assessed whether a non-linear boundary could improve discrimination using the same marker components. For each classifier family, the representative model reported in the main results was the configuration with the highest LOSO AUC.

#### 2.6.3 Deep-learning baseline models

Two end-to-end deep-learning baselines were implemented for reference: an EEGNet-style compact convolutional network (Lawhern et al., 2018) and a simple one-dimensional convolutional network (1D-CNN), both operating directly on pre-processed EEG epochs with input shape channels × time samples. Within each outer LOSO training set, three pre-specified configurations were evaluated for each architecture. Across the three EEGNet configurations, *F*_1_ ∈ {8, 16}, *F*_2_ ∈ {16, 32}, dropout ∈ {0.25, 0.50}, learning rate ∈ {3 × 10^−4^, 10^−3^}, and weight decay ∈ {10^−5^, 10^−4^}, using temporal kernels of 64 and 16 samples and depth multiplier *D* = 2. Across the three 1D-CNN configurations, base filters ∈ {8, 16, 32}, temporal kernel triplets (15, 9, 5) or (9, 7, 5), dropout ∈ {0.25, 0.40, 0.50}, and the same learning-rate and weight-decay ranges. Configuration selection used an inner subject-wise validation split; the configuration with the highest inner subject-level AUC was selected using data from the training subjects.

For the selected configuration, training used AdamW, batch size 64, a maximum of 80 epochs, and early stopping with patience 10. Channel-wise standardization was fitted on training epochs only. Training augmentation consisted of amplitude scaling (0.90–1.10), small Gaussian jitter (standard deviation 0.01–0.05 after standardization), and temporal circular shifts of up to 25 samples; validation and test epochs were left unchanged. Each participant contributed at most 56 clean training epochs per fold. Following the same LOSO protocol, models were trained at the epoch level using epochs from the training subjects only. Epoch-level predicted probabilities were then averaged into a single subject-level probability,


$$\begin{array}{l} {\hat{p}}_{i}=\frac{1}{m_{i}}\overset{m_{i}}{\underset{e=1}{\sum }}{\hat{p}}_{ie}, \end{array}$$ (15)

where *m*_*i*_ is the number of retained epochs for participant *i* and ${\hat{p}}_{ie}$ the predicted MDD probability for epoch *e*.

The searched EEGNet configurations contained 1,473–3,457 trainable parameters, whereas the searched 1D-CNN configurations contained 4,817–63,809 trainable parameters. Across the 48 outer folds, the selected EEGNet configurations were baseline, regularized augmentation, and larger augmentation in 22, 11, and 15 folds, respectively; the corresponding 1D-CNN counts were 16, 14, and 18 folds.

#### 2.6.4 Performance metrics and statistical analysis

Performance was evaluated at the subject level, with MDD as the positive class. Let TP, TN, FP, and FN denote true positives, true negatives, false positives, and false negatives. Accuracy, sensitivity, specificity, and F1-score were computed as


$$\begin{array}{l} \text{Accuracy}=\frac{\text{TP}+\text{TN}}{\text{TP}+\text{TN}+\text{FP}+\text{FN}}, \end{array}$$ (16)

$$\begin{array}{l} \text{Sensitivity}=\frac{\text{TP}}{\text{TP}+\text{FN}},\text{ Specificity}=\frac{\text{TN}}{\text{TN}+\text{FP}}, \end{array}$$ (17)

$$\begin{array}{l} \text{F}1=\frac{2\text{TP}}{2\text{TP}+\text{FP}+\text{FN}}. \end{array}$$ (18)

The area under the receiver operating characteristic curve (AUC) was computed from the continuous subject-level prediction scores (Hanley and McNeil, 1982).

A subject-level permutation test assessed whether the observed AUC exceeded chance-level discrimination, following the general framework of permutation testing for classifier performance (Ojala and Garriga, 2010). The LOSO prediction scores were held fixed while the subject labels were randomly permuted across participants, and the AUC was recomputed from the permuted labels and original scores. Repeating this *B* = 10, 000 times gave the empirical null distribution of AUC under random label assignment. The two-sided permutation *p* value compared the absolute deviation of the observed AUC from chance with the corresponding deviations of the null distribution:


$$\begin{array}{l} p_{\text{perm}}=\frac{1+\sum_{b=1}^{B}1\left\{\left|\text{AU}{\text{C}}_{\text{perm}}^{\left(b\right)}-0.5|\geq |\text{AU}{\text{C}}_{\text{obs}}-0.5\right|\right\}}{B+1}, \end{array}$$ (19)

where AUC_obs_ is the observed LOSO AUC and $\text{AU}{\text{C}}_{\text{perm}}^{\left(b\right)}$ the AUC from the *b*-th permutation. This evaluates whether the observed subject-level discrimination exceeds what would be expected under random association between scores and diagnostic labels.

A sensitivity power analysis was performed for the available cohort. For standardized between-group differences, the minimum detectable Cohen's *d* was calculated for a two-sided independent two-sample *t* test with 23 MDD participants, 25 HCs, α = 0.05, and 80% power using the non-central *t* distribution.

### 2.7 Model interpretability analysis

To interpret the selected marker-based classifier, SHAP (SHapley Additive exPlanations) analysis was applied to quantify the contribution of each marker component to the model output (Lundberg and Lee, 2017). SHAP provides an additive feature-attribution framework grounded in the Shapley value from cooperative game theory (Shapley, 1953): for a trained classifier *f* and a subject-level feature vector **x**_*i*_, the output is decomposed as


$$\begin{array}{l} f\left(x_{i}\right)=\phi_{0}+\overset{d}{\underset{j=1}{\sum }}\phi_{ij}, \end{array}$$ (20)

where ϕ_0_ is the baseline output, *d* the number of marker components, and ϕ_*ij*_ the contribution of feature *j* to the prediction for participant *i*. A positive SHAP value indicates a contribution toward the MDD decision, and a negative value indicates a contribution toward HC.

Kernel SHAP was applied to the decision function of the selected marker-based classifier under the same subject-wise LOSO framework used for classification (Lundberg and Lee, 2017). In each fold, the SHAP background set was constructed from the standardized marker features of the training subjects only, and SHAP values were computed for the held-out subject using out-of-fold predictions. Aggregating across folds, the global importance of each component was summarized by the mean absolute SHAP value,


$$\begin{array}{l} I_{j}=\frac{1}{n}\overset{n}{\underset{i=1}{\sum }}|\phi_{ij}|, \end{array}$$ (21)

where *n* is the number of participants; larger *I*_*j*_ indicates a larger average contribution to model predictions. In addition to global importance, subject-level SHAP values were examined as a function of each standardized feature value to assess the direction in which individual components shifted the decision toward MDD or HC.

## 3 Results

Before model analysis, two participants were excluded because fewer than 30 artifact-free epochs remained after quality control, yielding a final cohort of 48 participants (23 MDD and 25 HCs). All subsequent analyses were conducted at the subject level.

### 3.1 Exploratory feature patterns and marker definition

For the final cohort, 2494 subject-level EEG features were obtained without missing or infinite values, spanning spectral power, band-ratio, entropy and complexity, hemispheric asymmetry, and connectivity descriptors. This analysis characterized group-related feature patterns and identified a compact set of mechanism-relevant features for subsequent subject-wise validation. The univariate AUC values summarize exploratory feature-level discrimination.

**Figure 2:**
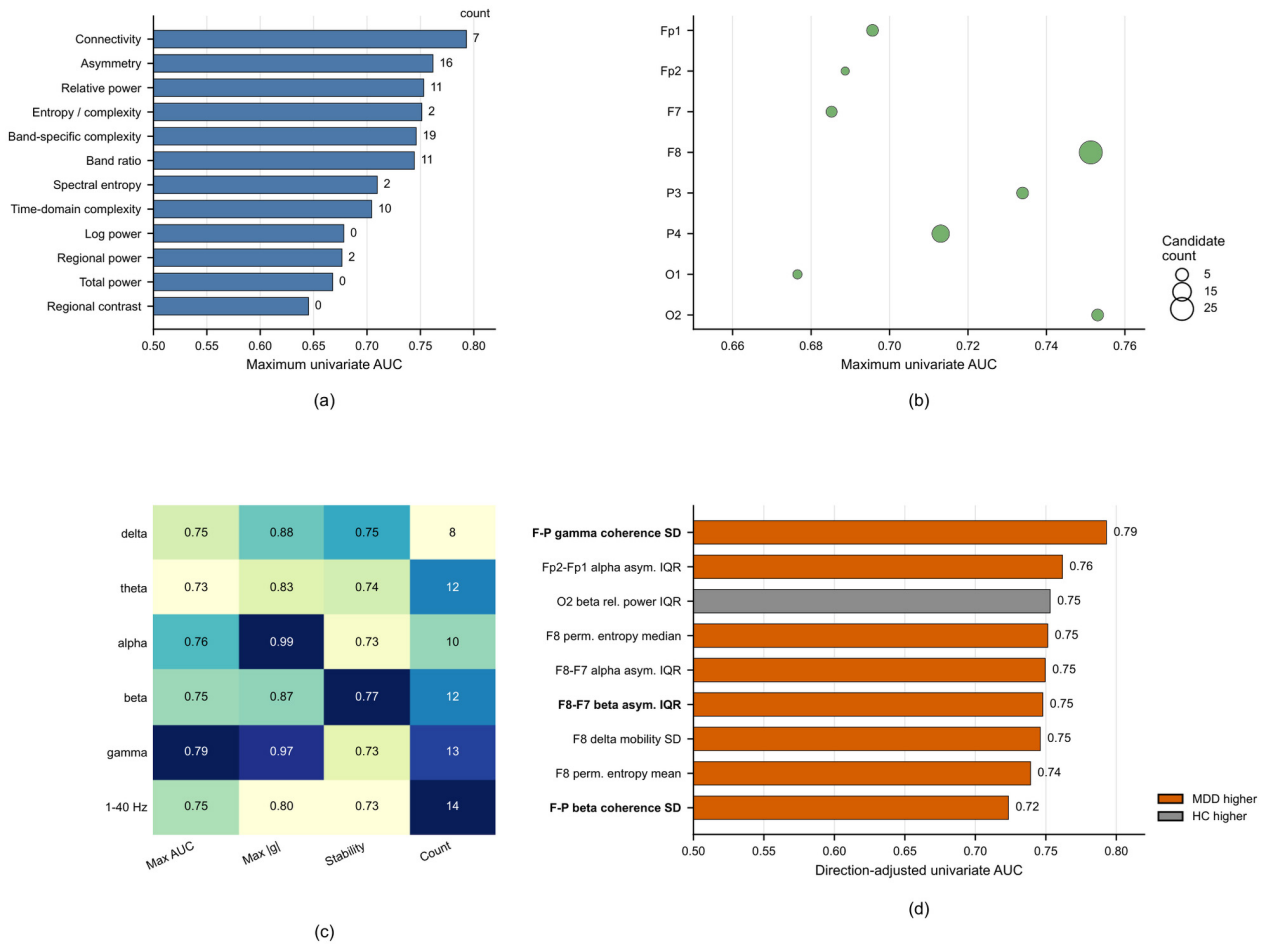
Exploratory feature analysis of subject-level EEG features. **(a)** Feature-family summary showing the maximum direction-adjusted univariate AUC within each feature family. Numbers at the end of the bars indicate the number of candidate features satisfying the predefined exploratory criteria. **(b)** Channel-level summary showing the maximum direction-adjusted univariate AUC for features assigned to each EEG channel. Bubble size represents the number of candidate features. **(c)** Frequency-band summary. The heatmap reports the maximum direction-adjusted univariate AUC, maximum absolute Hedges' *g*, bootstrap direction stability, and candidate-feature count for each frequency band. For visualization, heatmap colors were normalized within each metric column, whereas the numerical values shown in the cells are the original summary values. **(d)** Top exploratory features ranked by direction-adjusted univariate AUC. Orange bars indicate features with higher values in the MDD group, whereas gray bars indicate features with higher values in the HC group. Bold feature labels indicate components included in the proposed three-component EEG marker.

Across feature families (Figure 2a), connectivity features showed the highest maximum direction-adjusted univariate AUC, followed by asymmetry and relative-power features. Connectivity and asymmetry families also contributed multiple candidate features meeting the predefined exploratory criteria. Several power- and region-summary families showed weaker discrimination and fewer candidates. At the channel level, the right frontotemporal channel F8 stood out, contributing the largest number of candidate features assigned to it among the eight channels (Figure 2b). Among frequency bands, gamma showed the highest maximum direction-adjusted univariate AUC and beta the strongest bootstrap direction stability (Figure 2c). Alpha-band features also ranked among the stronger candidates, particularly for frontal asymmetry; nonetheless, marker definition was directed toward beta/gamma connectivity and beta asymmetry, as these were the most directly aligned with the fronto-posterior and right-frontotemporal mechanisms of interest.

The top-ranked exploratory features related mainly to fronto-posterior coherence heterogeneity, frontal asymmetry, and F8-associated complexity (Figure 2d). The single strongest feature was the gamma-band fronto-posterior coherence standard deviation, indicating that the spatial heterogeneity of fronto-posterior connectivity differed between groups. The F8–F7 beta-band asymmetry IQR was likewise among the leading MDD-higher features, supporting the relevance of right frontotemporal asymmetry variability. The beta-band fronto-posterior coherence standard deviation showed a lower univariate AUC than its gamma-band counterpart but was retained, as it captures the same fronto-posterior connectivity mechanism in a complementary band.

On this basis, we defined a compact three-component EEG marker comprising (i) fronto-posterior beta-band coherence spatial heterogeneity, (ii) fronto-posterior gamma-band coherence spatial heterogeneity, and (iii) within-subject variability of F8–F7 beta-band log-power asymmetry. The first two components are the standard deviation of band-specific coherence across frontal–posterior channel pairs, and the third is the interquartile range of F8–F7 beta-band log-power asymmetry across retained epochs. This marker was then fixed and evaluated under strict subject-wise LOSO validation.

### 3.2 Marker-based classification under subject-wise LOSO validation

Using the proposed three-component EEG marker as the common input, we evaluated classification performance under strict subject-wise LOSO validation across four classifier families: ridge logistic regression, linear SVM, shrinkage LDA, and RBF-SVM. All classifiers used the same fixed three-component subject-level marker, and for each family the configuration with the highest LOSO AUC is reported.

**Figure 3:**
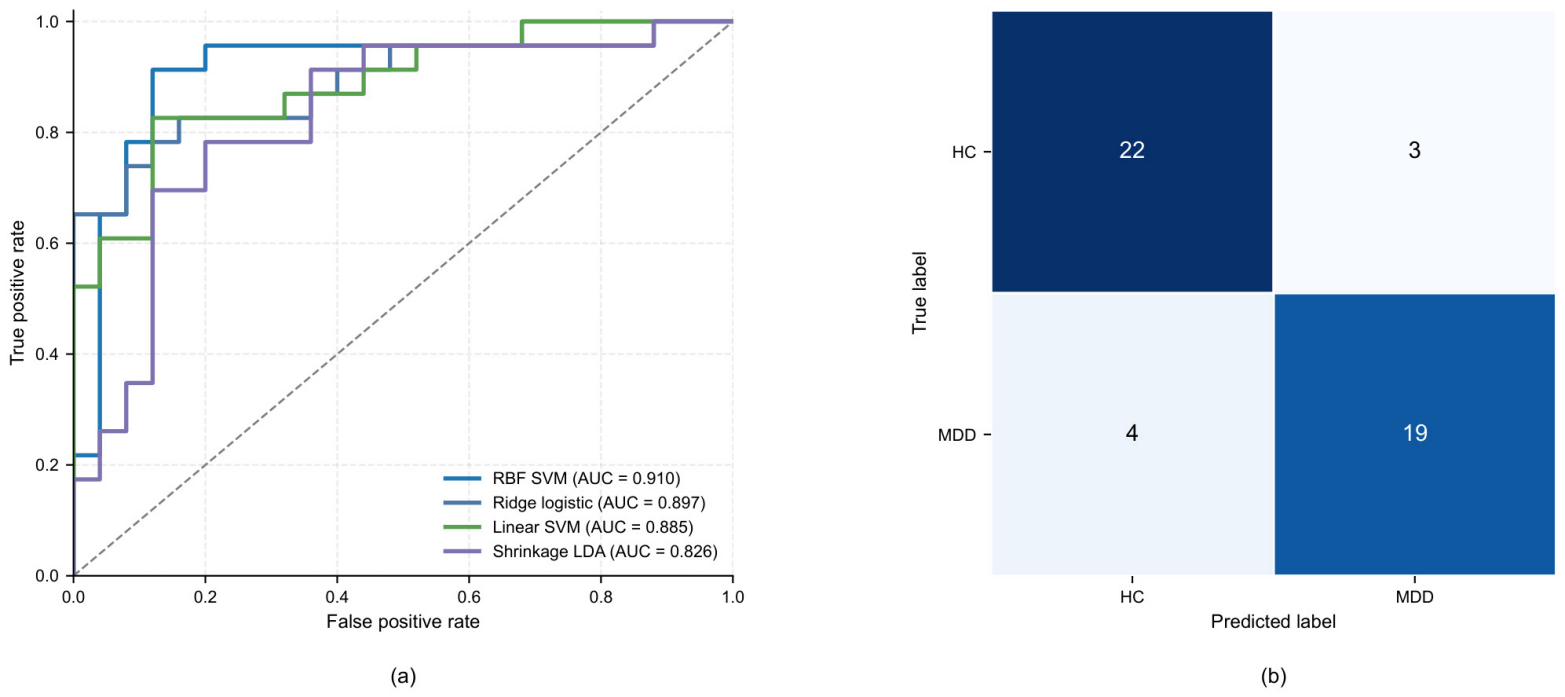
Marker-based classifier performance under subject-wise LOSO validation. All classifiers used the same proposed three-component EEG marker as input. **(a)** Receiver operating characteristic curves for the representative model within each classical classifier family, taken as the configuration with the highest LOSO AUC. The legend reports the corresponding AUC values. **(b)** Confusion matrix of the RBF-SVM classifier, which achieved the highest LOSO AUC among the evaluated marker-based classifiers. Predictions were obtained using the default SVM decision-score threshold. HC and MDD denote healthy controls and major depressive disorder, respectively.

Table 2 summarizes the subject-level performance. RBF-SVM attained the highest AUC (0.910), with an accuracy of 85.42%, sensitivity of 82.61%, specificity of 88.00%, and F1-score of 84.44%. Notably, the linear models were closely competitive: linear SVM matched RBF-SVM exactly on all threshold-dependent metrics (accuracy, sensitivity, specificity, and F1) and differed only in AUC (0.885), while ridge logistic regression reached an AUC of 0.897 with an accuracy of 83.33%. This pattern suggests that much of the discriminative information in the marker was also captured by linear decision rules, although RBF-SVM provided the highest threshold-independent discrimination. Shrinkage LDA was weaker (AUC 0.826, accuracy 70.83%). The ROC curves in Figure 3a show the same ordering, with RBF-SVM giving the strongest threshold-independent discrimination. Its confusion matrix (Figure 3b) correctly identified 22 of 25 HCs and 19 of 23 MDD subjects, with three false positives and four false negatives. Within this internal subject-wise LOSO validation, RBF-SVM was thus the best-performing marker-based classifier, while remaining closely comparable to the linear models.

**Table 2:** Subject-wise LOSO performance of marker-based classifiers using the proposed three-component EEG marker.

Classifier	Accuracy (%)	Sensitivity (%)	Specificity (%)	F1-score (%)	AUC
RBF-SVM	**85.42**	**82.61**	**88.00**	**84.44**	**0.910**
Linear SVM	**85.42**	**82.61**	**88.00**	**84.44**	0.885
Ridge logistic regression	83.33	**82.61**	84.00	82.61	0.897
Shrinkage LDA	70.83	69.57	72.00	69.57	0.826

For each classifier family, the configuration with the highest LOSO AUC is shown. The best value for each performance metric is shown in bold.

**Figure 4:**
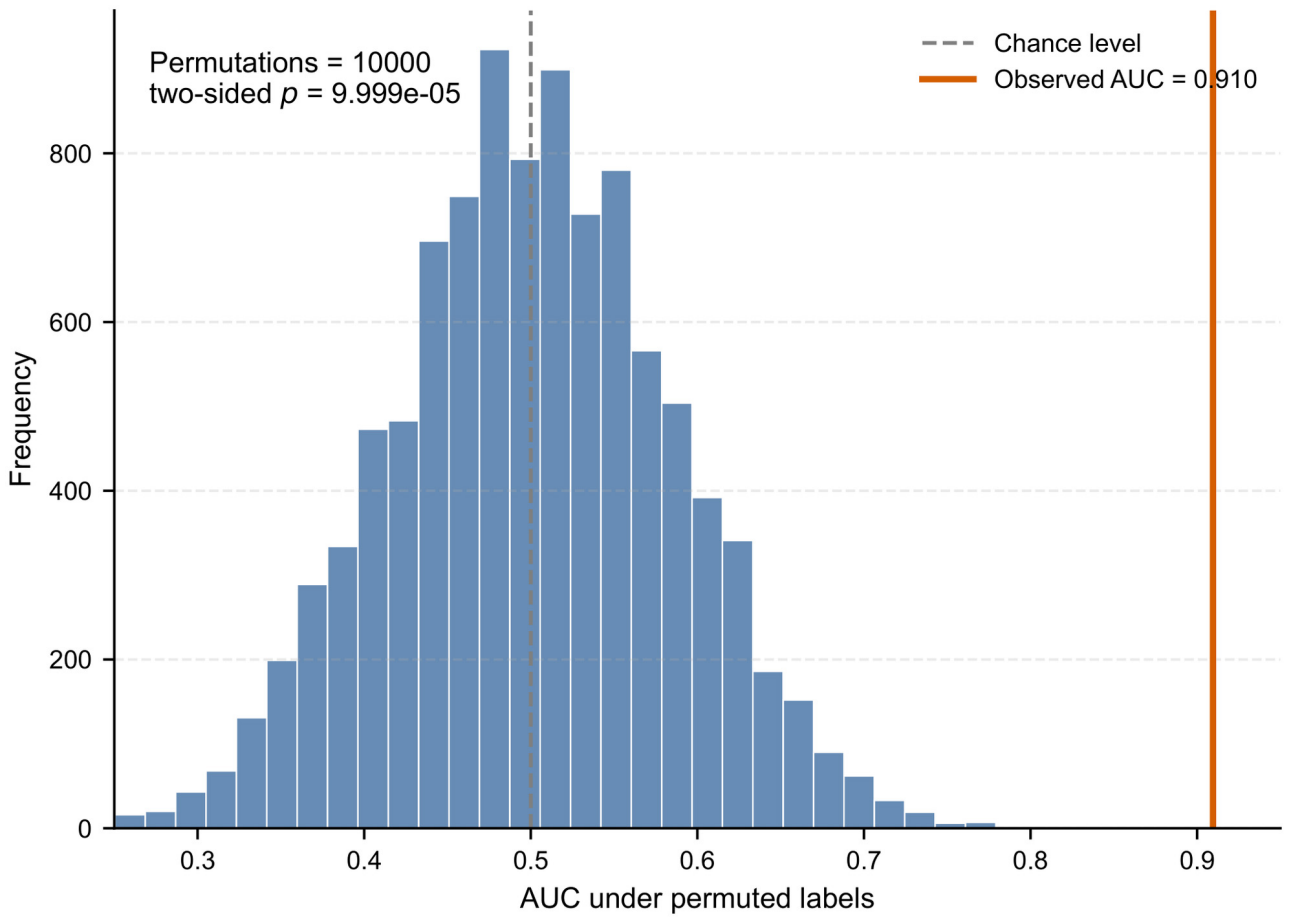
Subject-level permutation test for the selected RBF-SVM classifier. The histogram shows the null distribution of AUC values obtained by randomly permuting the subject labels while keeping the LOSO prediction scores fixed. The dashed vertical line indicates chance-level discrimination (AUC = 0.5), and the solid vertical line indicates the observed AUC of the selected RBF-SVM classifier. The observed AUC was clearly separated from the permutation distribution, supporting above-chance subject-level discrimination.

To assess whether this discrimination exceeded chance, a subject-level permutation test was applied to the selected RBF-SVM classifier: the LOSO prediction scores were held fixed while the subject labels were permuted 10,000 times. As shown in Figure 4, the permuted-AUC null distribution was centered near chance, whereas the observed AUC of 0.910 lay clearly outside it (two-sided *p* < 0.001), supporting above-chance subject-level discrimination.

The sensitivity power analysis indicated that, with the available 23 MDD participants and 25 HCs, the minimum standardized between-group difference detectable at 80% power and a two-sided α = 0.05 was Cohen's *d* = 0.827.

### 3.3 Comparison with deep-learning baselines

To contextualize the marker-based results, we compared the selected RBF-SVM classifier with two end-to-end deep-learning baselines, EEGNet and a simple 1D-CNN, evaluated under the same subject-wise LOSO protocol; for these baselines, models were trained on epochs from the training subjects and epoch-level probabilities were averaged into subject-level predictions. As shown in Table 3, the marker-based RBF-SVM classifier exceeded both tuned baselines on the primary subject-level metrics (accuracy 85.42%, sensitivity 82.61%, specificity 88.00%, F1-score 84.44%, AUC 0.910). With a common 0.5 threshold, tuned EEGNet reached an accuracy of 41.67% and AUC of 0.456, while the tuned 1D-CNN reached an accuracy of 45.83% and AUC of 0.433. Selecting the decision threshold from the inner validation set increased EEGNet accuracy to 54.17% and 1D-CNN accuracy to 41.67%; their threshold-independent AUCs remained 0.456 and 0.433, respectively. The evaluated end-to-end convolutional baselines showed limited subject-wise discrimination under the LOSO evaluation.

**Table 3:** Subject-wise LOSO performance of the proposed marker-based classifier and tuned deep-learning baselines.

Method	Accuracy (%)	Sensitivity (%)	Specificity (%)	F1-score (%)	AUC
Proposed (RBF-SVM)	**85.42**	**82.61**	**88.00**	**84.44**	**0.910**
EEGNet (tuned)	41.67	56.52	28.00	48.15	0.456
1D-CNN (tuned)	45.83	47.83	44.00	45.83	0.433

The proposed method used the three-component EEG marker with RBF-SVM, whereas tuned EEGNet and 1D-CNN were trained on pre-processed EEG epochs and evaluated by subject-level probability aggregation. Deep-learning values use the common 0.5 decision threshold; inner-validation threshold results are reported in the text. The best value for each performance metric is shown in bold.

### 3.4 SHAP interpretation of the selected RBF-SVM classifier

To interpret the selected marker-based RBF-SVM classifier, Kernel SHAP was applied to its decision function under the same subject-wise LOSO framework. SHAP values were computed for held-out subjects using out-of-fold predictions. Positive SHAP values denote contributions toward the MDD decision score and negative values toward HC.

**Figure 5:**
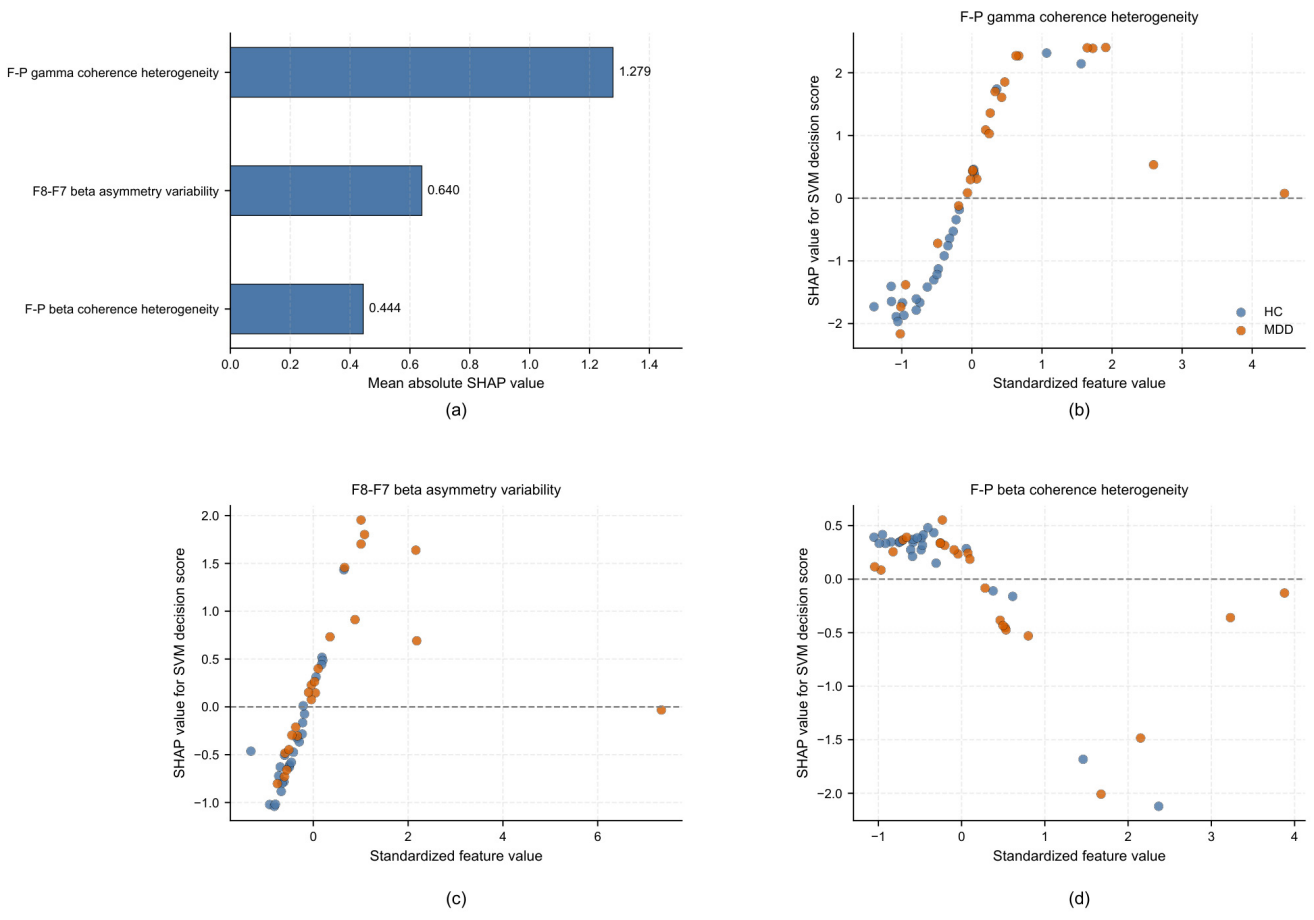
SHAP interpretation of the selected marker-based RBF-SVM classifier. Kernel SHAP was applied to the RBF-SVM decision function under the subject-wise LOSO framework. Positive SHAP values indicate contributions toward the MDD decision score, whereas negative SHAP values indicate contributions toward the HC decision score. **(a)** Global SHAP importance of the three marker components, quantified by the mean absolute SHAP value across held-out subjects. **(b–d)** Feature-wise SHAP dependence plots for fronto-posterior gamma-band coherence heterogeneity, F8–F7 beta-band asymmetry variability, and fronto-posterior beta-band coherence heterogeneity, respectively. The horizontal dashed line marks zero SHAP contribution. Points are colored according to the true diagnostic group. F-P denotes fronto-posterior.

Globally (Figure 5a), fronto-posterior gamma-band coherence heterogeneity had by far the largest mean absolute SHAP value and was the dominant contributor to the decision function, followed by F8–F7 beta-band asymmetry variability, with fronto-posterior beta-band coherence heterogeneity making a smaller but non-negligible contribution. The dependence plots show the direction of these effects. For fronto-posterior gamma-band coherence heterogeneity, higher standardized values were associated with positive SHAP values and lower values with negative ones, so that greater gamma-band coherence heterogeneity shifted the decision score toward MDD (Figure 5b). F8–F7 beta-band asymmetry variability showed a similar pattern, with larger values associated with higher MDD decision scores (Figure 5c). Fronto-posterior beta-band coherence heterogeneity, by contrast, had lower overall importance and a more complex, mixed-sign relationship across its range (Figure 5d).

Overall, the SHAP analysis indicates that the classifier separated MDD from HC mainly through fronto-posterior gamma-band coherence heterogeneity and F8–F7 beta-band asymmetry variability, with fronto-posterior beta-band coherence heterogeneity providing complementary non-linear information. These results support the interpretability of the proposed marker by linking the model decision function to specific connectivity and asymmetry patterns at the classifier level.

## 4 Discussion

In this study, we developed and internally evaluated an interpretable machine-learning framework for depression classification based on a compact three-component EEG marker. The marker comprised subject-level components derived from fronto-posterior coherence heterogeneity and right frontotemporal beta-band asymmetry variability, which emerged from exploratory feature analysis. Using this marker as a common input, marker-based classical classifiers achieved above-chance subject-level discrimination under strict subject-wise LOSO validation, whereas the evaluated end-to-end convolutional baselines did not achieve reliable subject-wise generalization in this small-sample, eight-channel cohort. A subject-level label-permutation test supported that the observed discrimination was unlikely to arise under random label assignment. SHAP analysis further linked the selected classifier's decisions to specific coherence and asymmetry components, providing an interpretable account of how the marker contributed to the model output.

The three marker components are broadly consistent with neurophysiological patterns previously associated with depression. The two coherence components quantify the spatial heterogeneity of fronto-posterior coherence in the beta and gamma bands, in line with reports of altered EEG functional connectivity and disrupted long-range coordination in MDD (Leuchter et al., 2012; Miljevic et al., 2023). The gamma-band component is also consistent with evidence implicating gamma oscillatory abnormalities in depression (Fitzgerald and Watson, 2018). The third component, the within-subject variability of F8–F7 beta-band asymmetry, echoes the literature on right frontotemporal and frontal asymmetry in affective regulation (Allen and Reznik, 2015; Luo et al., 2025). The SHAP analysis was consistent with these associations: fronto-posterior gamma-band coherence heterogeneity was the dominant contributor, with higher values shifting the decision toward MDD, and F8–F7 beta-band asymmetry variability contributed in the same direction. These correspondences provide a classifier-level interpretation of the group-related EEG patterns and motivate targeted neurophysiological investigation.

The value of the marker-based strategy was most apparent in comparison with the evaluated end-to-end deep-learning baselines. In this small-sample, eight-channel cohort, the tuned convolutional models did not achieve reliable subject-wise generalization under LOSO, consistent with the known difficulty of training end-to-end EEG models that generalize across subjects when data are limited (Roy et al., 2019; Xu et al., 2020). Although the deep-learning models were trained on multiple epochs, the number of statistically independent units was still determined by the number of subjects. The present tuning analysis evaluated changes in model capacity, regularization, and training-only augmentation. Within the present small-sample, eight-channel, subject-wise LOSO setting, the compact marker-based models generalized more effectively than the evaluated EEGNet and 1D-CNN baselines. Larger cohorts can support evaluation of broader architectures and augmentation policies.

The interpretability of the proposed framework also clarifies how the selected classifier used the marker. Because the marker contains only three components, its contribution to the decision can be examined directly. Moreover, although RBF-SVM achieved the highest threshold-independent discrimination, the linear classifiers were closely competitive, suggesting that much of the discriminative information in the marker was accessible to relatively simple decision rules. Taken together, the compact feature space, the SHAP-based attribution pattern, and the comparable performance of linear and non-linear classifiers suggest that the observed discrimination was mainly driven by a small set of interpretable EEG components.

Several limitations should be considered when interpreting these results. First, the sample was small (48 participants) and drawn from a single center. The sensitivity power analysis indicated that the available sample could detect a standardized between-group difference of Cohen's *d* = 0.827 with 80% power, suggesting that smaller effects may not be reliably detected and limiting the generalizability of the findings. Second, the targeted low-channel montage sampled bilateral frontal and posterior regions but did not include Fz, Cz, or Pz. Accordingly, the reported fronto-posterior coherence should be interpreted as a low-density, sensor-level connectivity summary between predefined frontal and posterior electrode sets, rather than as complete whole-brain or source-level connectivity. Because coherence estimates may depend on the reference configuration, the findings should be interpreted within the bilateral A1/A2 mastoid-reference setting. Third, the 2-s epoch choice may yield less stable coherence estimates than longer recordings or multitaper spectral procedures, although previous resting-state EEG depression studies have used 2-s segments for spectral and connectivity analysis. Medication information and structured assessments of psychiatric comorbidities were unavailable; future validation studies should incorporate these variables as pre-specified clinical covariates. Finally, although every held-out subject was excluded from model fitting, feature standardization, validation, and early stopping within each LOSO fold, the marker components and classifier configurations were established on the same cohort. The reported performance should therefore be regarded as internal subject-wise validation rather than confirmation of a clinically deployable diagnostic tool. The absence of an independent external cohort means that the stability and transferability of the marker-based framework remain to be established.

## 5 Conclusion

This study developed a three-component EEG marker-based interpretable machine-learning framework using eight-channel resting-state EEG in a single-center pilot cohort. Under strict subject-wise LOSO validation, the proposed marker supported above-chance subject-level discrimination in the primary native bilateral A1/A2 mastoid-reference analysis. SHAP analysis indicated that the selected classifier primarily relied on fronto-posterior gamma-band coherence heterogeneity and F8–F7 beta-band asymmetry variability, supporting the interpretability of the marker-based decision process within this analysis setting. These findings suggest that compact subject-level EEG markers may be useful for hypothesis generation in data-limited EEG studies. Future work in larger, independent, and multi-center cohorts with standardized clinical characterization, higher-density EEG recordings, predefined referencing schemes, and longitudinal follow-up will evaluate the reproducibility, transferability, and clinical relevance of the framework.

## Data Availability

The raw data supporting the conclusions of this article will be made available by the authors, without undue reservation.
